# Highly Transparent, Flexible and Conductive CNF/AgNW Paper for Paper Electronics

**DOI:** 10.3390/ma12020322

**Published:** 2019-01-21

**Authors:** Ren’ai Li, Kaili Zhang, Guangxue Chen

**Affiliations:** State Key Laboratory of Pulp & Paper Engineering, South China University of Technology, Guangzhou 510640, China; nalirix@163.com (R.L.); zkl152712@163.com (K.Z.)

**Keywords:** conductive paper, cellulose nanofiber, silver nanowires, choline chloride/urea

## Abstract

Conductive paper has the advantages of being low-cost, lightweight, disposable, flexible, and foldable, giving it promising potential in future electronics. However, mainstream conductive papers are opaque and rigid, which seriously affect the wide application of conductive paper. In this paper, we demonstrate a highly transparent, flexible, and conductive paper, fabricated by mixing cellulose nanofibers (CNFs) with silver nanowires (AgNWs) and then plasticizing with choline chloride/urea solvent. The as-prepared CNF/AgNW paper showed high transparency (~90% transmittance) and flexibility (~27% strain), and low sheet resistance (56 Ω/sq). Moreover, the resistance change of CNF/AgNW paper increased only ~1.1% after 3000 bending−unbending cycles under a 150° large angle, implying a long working life and stability. In view of this, our methodology has the potential to open a new powerful route for fabrication of paper-based green electronics.

## 1. Introduction

With the rapid development of the electronics industry, electronic components with flexibility, low cost, and green features are the developing trend. Meanwhile, the waste associated with traditional electronic components is causing environmental pollution [1]. Therefore, it is imperative to find suitable materials to solve these problems. As an old and widely-used material, paper has garnered considerable interest due to its advantages of being lightweight, portable, flexible, foldable, biodegradable, low-cost, and occupying a small amount of space. All these features bestow paper the potential to be used in future electronic fields [2]. Paper is mainly composed of natural cellulose. High surface roughness, porous structure, and optical opaqueness of raw paper are intrinsic barriers to paper’s potential roles in electronic components. As an insulating material, paper’s resistivity and square resistance are around 10^8^–10^12^ Ω·m and 10^11^–10^15^ Ω·sq^-1^, respectively. In order to re-engineer paper to be conductive, Graphene [3,4,5,6,7], carbon fiber [8,9,10,11], conductive carbon black [12], graphite [13], conductive polymers [14,15,16,17], and metal powder [18] all have been used to prepare a paper-based conductive composite. The paper-based conductive materials can be widely used in batteries [19,20], transistors [21], supercapacitors [7], solar cells [22], sensors [23,24,25,26,27], actuators [28], etc. However, the conductive fillers in the composite network can easily be oxidized or fall off [29], which gives the conductive paper a low conductivity, and also a high Young’s modulus (10^9^~10^11^ Pa) [30] and unsatisfactory transparency (opaque or totally black) [29]. These problems greatly limit wide application of conductive paper. Moreover, the integration of highly transparent, flexible paper with stable conductivity remains a challenge.

**Figure 1:**
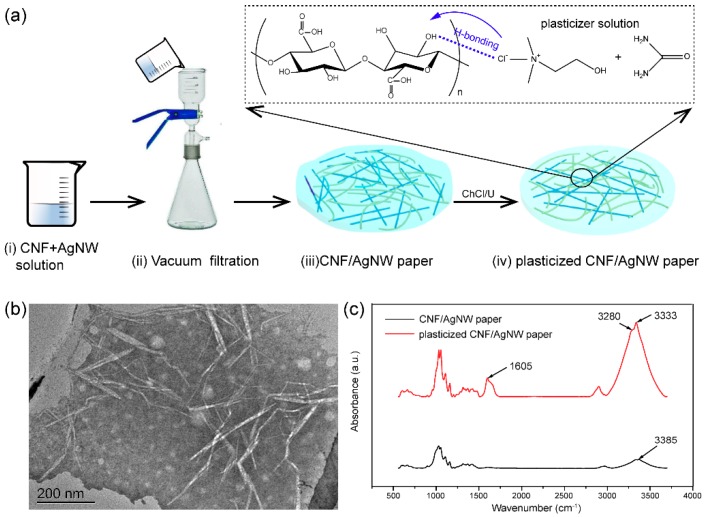
Fabrication process and characterization of plasticized conductive paper. (**a**) Preparing conductive paper includes: (i) mixing the CNFs with AgNWs thoroughly; (ii) vacuum filtration of CNF/AgNW mixed solution; (iii) the CNF/AgNW hybrid gels; and (iv) the plasticized transparent, flexible, conductive CNF/AgNW paper. (**b**) TEM image of CNF. (**c**) FTIR spectra of CNF/AgNW paper and plasticized CNF/AgNW paper.

Herein, we demonstrate a highly transparent, flexible, and conductive cellulose nanofiber/silver nanowire (CNF/AgNW) paper. The CNF/AgNW paper was prepared by mixing these two components and then plasticizing with choline chloride/urea (ChCl/U) mixture. The fabrication process was shown in Figure 1. ChCl/U contributed two significant advantages in the fabrication process: (1) ChCl/U is a green, low-cost, eco-friendly, and recycled solvent which is simple to prepare [31]; (2) ChCl/U, as an effective plasticizer [32,33], bestows the CNF/AgNW paper a low Young’s modulus, and high transparency and flexibility. The as-prepared CNF/AgNW paper has low sheet resistance and negligible resistance increase after 3000 bending–unbending cycles. Our methodology has the potential to fabricate transparent, flexible, and conductive paper for applications in future paper-based electronics. 

## 2. Materials and Methods 

### 2.1. Materials

Choline chloride (ChCl, 98%, Shanghai Macklin Biochemical Co., Ltd, Shanghai, China), urea (U, AR, 99%, Macklin, Shanghai, China), 2,2,6,6-tetramethylpiperidine-1-oxyl radical (TEMPO, 98%, Macklin, Shanghai, China ), NaOH (AR, 95%, Macklin, Shanghai, China), NaClO (AR, available chlorine 4.0%, Macklin, Shanghai, China), NaBr (AR, 99%, Macklin, Shanghai, China), HCl (AR, GHtech, Guangzhou, China), ethanol (AR, 99.7%, Macklin, Shanghai, China) and silver nanowires (AgNWs, 0.1 mg/ml, Shanghai Aladdin agent, Shanghai, China) were used as received. 

### 2.2. Preparation of TEMPO-oxidized CNFs

CNF was synthesized according to previous literature [34,35,36]. TEMPO (0.157 g, 0.001 mol) and NaBr (1 g, 0.01 mol) were added to 10 g cellulose aqueous suspensions (solid content: 1%). An 11 wt.% NaClO solution was adjusted to pH 10 by the addition of 0.1 M HCl. TEMPO-mediated oxidation of CNF was initiated by adding a desired amount of NaClO solution (10 mmol per gram of cellulose) and was continued at room temperature under constant stirring for 4 h. The pH was kept at 10 using 0.5 M NaOH monitored by a pH meter. After the reaction, 10 mL (1 wt.%) of ethanol is added to terminate the reaction. The water-insoluble fraction was recovered by centrifugation and washed thoroughly with water 3~5 times. As shown in Figure 1b, the length–diameter ratio was about 50:1. The solid content of CNF was measured to 0.5 wt.%. 

### 2.3. Fabrication of Conductive Paper 

TEMPO-oxidized CNFs were used to prepare CNF/AgNW paper. AgNWs were mixed in an aqueous suspension of CNFs with various concentrations (the mass ratio of AgNWs to CNFs were 0:100, 0.5:100, 1.0:100, 1.5:100, 2.0:100, and 2.5:100, respectively), followed by vacuum filtration. The obtained CNF/AgNW hybrid gels were soaked in the as-prepared ChCl/U aqueous solutions for 12 h. The ChCl/U (molar ratio of 1:2) solution with molar ratio of ChCl/U to H_2_O (0.04:1) was prepared by adding desired amount U and ChCl into deionized water. Then, the CNF/AgNW gels were dried by hot pressing at 60 °C for 10 h under the pressure of 10 MPa, and finally were peeled off from the filter to obtain plasticized CNF/AgNW paper. The reference sample without ChCl/U was also prepared in a similar way to above.

## 3. Characterizations

Fourier transform infrared (ATR-FTIR) spectra were recorded on a Bruker Vertex 33 spectrometer. The dispersion state of CNF suspension was examined with transmission electron microscopy (TEM, JEM-2010, JEOL). A UV-visible spectrometer (Cary60, Agilent, USA) was applied to test the regular light transmittance of conductive paper. The wavelength range is 200–1000 nm with a speed of 600 nm/min. Scanning electron microscope (SEM) images were obtained by using the HITACHI TM3030 Tabletop SEM. The tensile testing was performed using a tensile machine (INSTRON 5565, 100N load cell, tensile speed = 1 mm/min). The sheet resistance of conductive paper was measured by using the four-point probe technique (ST2258A, measuring range: straight line 2 + 2 + 2 mm pitch, sensitivity: ± 0.5%, detection limit: 1000 g, Suzhou Jingge Electronic). The bending−unbending cycles were manipulated with the assistance of a translation stage (Model LTS150/M, Thorlabs), and the frequency was set to 0.5 Hz. Optical images were taken by a Nikon Digital Sight DS-Fil camera (D750, Nikon Corporation, Tokyo, Japan). 

## 4. Results

**Figure 2:**
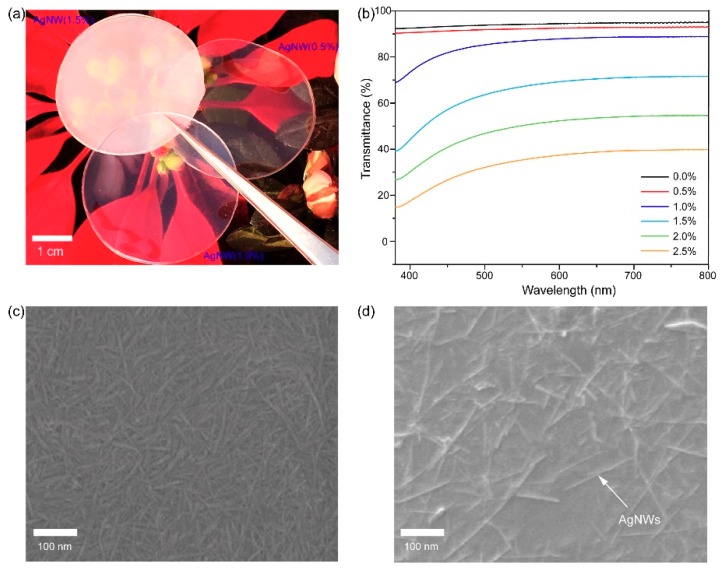
Optical properties of conductive paper: (**a**) Digital photograph of conductive paper with various AgNW contents; (**b**) optical transmittance of conductive paper; (**c**) SEM images of pristine CNF paper; (**d**) plasticized CNF/AgNW paper.

Though tremendous work has been done exploring conductive cellulose paper, the rigid features due to the extensive hydrogen bonds among cellulose chains and unsatisfied optical performances are the problems that restrict their wide application. In this experiment, CNFs are selected as substrate because of their high strength, transparency, renewability, biodegradability and abundance in nature. AgNWs are ideal to prepare conductive materials because of their excellent electrical conductivity. Firstly, CNFs and AgNWs were mixed with different weight ratios (100:0.5, 100:1.0, 100:1.5, 100:2.0, 100:2.5), and then the hybrid gels, after vacuum filtration, were plasticized in ChCl/U solvent for hours. Finally, we obtained the plasticized CNF/AgNW paper. Figure 2a exhibited photographs of CNF papers that contained different AgNW contents. The AgNW content has a great influence on the transparency of the paper. As shown in Figure 2b, the optical transmittance gets up to ~90% when the AgNW content is 0.5 wt.%. As the AgNW content increased, the transparency dropped sharply because of the scattering of AgNWs to visible light. In addition, the multiplied opaque AgNWs also blocked the light transmittance and reduced transparency. The SEM image of pristine CNF paper shows that the bare nanofibers were closely entangled, making CNF paper a rough surface (Figure 2c), while the surface topography of the plasticized CNF/AgNW paper was much smoother than the pristine CNF paper. The distribution of AgNWs on the paper surface indicates their successful introduction into the conductive paper network (Figure 2d).

**Figure 3:**
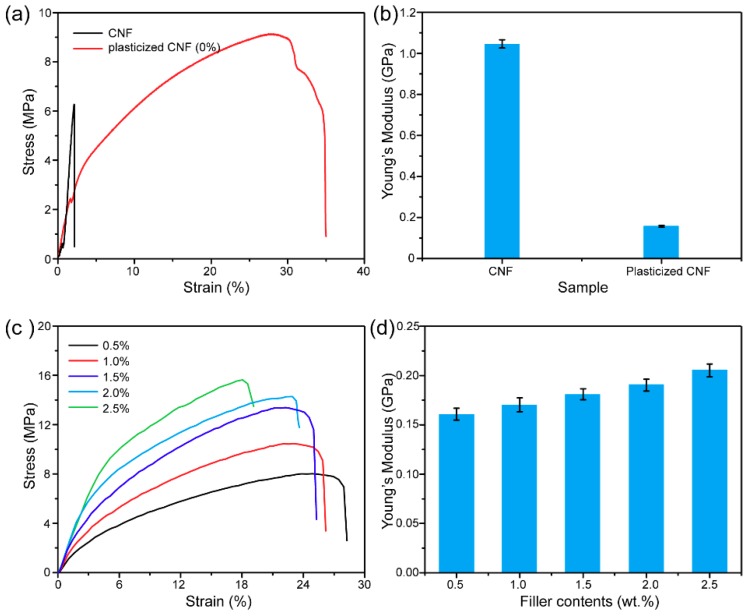
Mechanical properties of CNF/AgNW conductive paper: (**a**) The strain–stress curve for the CNF paper and plasticized CNF paper; (**b**) Young’s modulus of CNF paper and plasticized CNF paper; (**c**) the strain–stress curve for the plasticized CNF/AgNW paper; (**d**) Young’s modulus of plasticized CNF/AgNW paper.

The effect of ChCl/U plasticization on the mechanical properties of CNF paper was shown in Figure 3a. The tensile strain of pristine CNF paper is only about 2%. As discussed previously, CNF is rigid and brittle, a poor mechanical property. In contrast, the tensile deformation of the plasticized CNF paper was greatly improved, and the strain was ~35% (Figure 3b). The reason for this may be that the Cl^−^ anion of ChCl tends to detach the hydrogen atom in -OH in cellulose chains, and the choline cation may interact with the oxygen atom. Urea molecules form a complex with ChCl through the N–H···Cl hydrogen bond, and ChCl acts as a bridge between the urea and cellulose moieties [37]. As shown in Figure 1c, new absorption bands at around 3280 and 1605 cm^−1^ appeared in the spectra of plasticized CNF/AgNW paper, which are the characteristic bands of ChCl/U. Noticeably, the bands of the O–H stretching of alcohol group and the N–H stretching vibration of amide group shifted to lower wavenumbers. As a result, the intramolecular hydrogen bond among cellulose chains were greatly weakened, making the paper soft and flexible. However, due to the introduction of AgNWs in the paper network, the tensile properties decreased overall (Figure 3c). This may be because the addition of AgNWs had a side effect on the hydrogen bonds formed between ChCl and cellulose. Figure 3d showed that the Young’s modulus of the plasticized CNF/AgNW paper was slightly raised with the increasing AgNW content, because of the rigid feature of AgNWs. 

**Figure 4:**
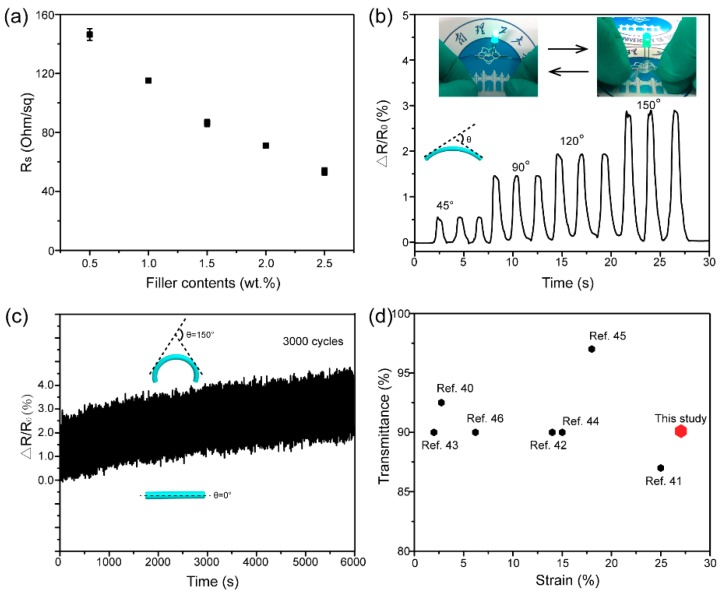
Electrical properties of CNF/AgNW conductive paper: (**a**) Sheet resistances of conductive paper; (**b**) plots of resistance change of conductive paper as a function of time for the bending angle in the range of 45−150°; (**c**) lifetime test at a bending angle of 150° (frequency: 0.5 Hz)—the resistance change curves were recorded for 3000 cycles; (**d**) comparison of strain and transmittance of this study with other transparent, flexible, and conductive paper.

In addition to mechanical properties, CNF/AgNW paper also displayed excellent electrical properties. The sheet resistance of pristine CNF paper was too large to measure, while with the addition of AgNWs, the sheet resistance decreased from 143 Ω/sq to 56 Ω/sq (Figure 4a). Due to electrical conductivity, CNF/AgNW paper could detect the resistance change caused by paper deformation. As shown in Figure 4b, the resistance change of CNF/AgNW paper was continually increasing, with the bending angle ranging from 45° to 150°. The inset picture illustrates its conductivity and bendability. The electrical cycling stability and bend-resistance ability, critical parameters in the flexible substrates, were tested under a 150° large bending angle (Figure 4c). The response of resistance change, compared to initial resistance with the bending applied on CNF/AgNW paper, increased only ~1.1% after 3000 bending−unbending cycles, implying a long working life and stability. It is worth mentioning that the as-prepared conductive paper has higher transparency than the current mainstream carbon-based conductive paper [4,7,20,38,39]. In particular, high transparency will allow it to transmit electrical signals without impeding optical signal when applied to electrochromic devices, touch sensors, solar cells, transistors, organic light-emitting diodes, etc. Compared with the current transparent conductive paper [40,41,42,43,44,45,46] as shown in Figure 4d, our prepared CNF/AgNW paper showed better mechanical performance (~ 27% strain).

## 5. Conclusions

To summarize, this work introduced a novel and facile way to prepare transparent, flexible, conductive paper, which used CNFs as substrates and AgNWs as conductive components to ensure the electrical conductivity. To enhance the mechanical properties, CNF/AgNW paper was plasticized in ChCl/U solvent. As a result, the as-prepared CNF/AgNW paper showed high transparency (~90% transmittance) and flexibility (~27% strain), and low sheet resistance. Moreover, there was only a ~1.1% resistance increase compared to the initial resistance after 3000 cycles of bending–unbending under a 150° large angle, implying a long working life and excellent cycle stability. The cellulose is renewable and abundant in nature, and the fabrication process is simple and green, which will further promote the application of paper-based conductive materials in flexible electronic devices, solar cells, transparent touch panels, etc.
